# Interaction between lifestyle and genetic susceptibility in myopia: the Generation R study

**DOI:** 10.1007/s10654-019-00512-7

**Published:** 2019-04-03

**Authors:** Clair A. Enthoven, Jan Willem Lodewijk Tideman, Jan Roelof Polling, Milly S. Tedja, Hein Raat, Adriana I. Iglesias, Virginie J. M. Verhoeven, Caroline C. W. Klaver

**Affiliations:** 1 Department of Ophthalmology, Erasmus University Medical Centre, Room Na-2808, PO Box 2040, 3000 CA Rotterdam, The Netherlands; 2 Department of Epidemiology, Erasmus University Medical Centre, Rotterdam, The Netherlands; 3 The Generation R Study Group, Erasmus University Medical Centre, Rotterdam, The Netherlands; 4 Department of Public Health, Erasmus University Medical Centre, Rotterdam, The Netherlands; 5 Department of Orthoptics & Optometry, University of Applied Sciences, Utrecht, The Netherlands; 6 Department of Clinical Genetics, Erasmus University Medical Centre, Rotterdam, The Netherlands; 7 Department of Ophthalmology, Radboud University Medical Centre, Nijmegen, The Netherlands

**Keywords:** Myopia, Refractive error, G×E, Gene-environment, Environmental factors, Children

## Abstract

Myopia is a refractive error of the eye caused by a complex interplay between nature and nurture. The aim of this study was to investigate whether environmental risk factors can influence the genetic effect in children developing myopia. A total of 3422 children participating in the birth-cohort study Generation R underwent an extensive eye examination at 9 years with measurements of refractive error and axial length corneal radius ratio (AL/CR). Environmental risk factors were evaluated using a questionnaire, and environmental risk scores (ERS) were calculated using backward regression analyses. Genetic risk scores (GRS) were calculated based on all currently known risk variants for myopia. Gene-environment interaction (G×E) was investigated using linear and logistic regression analyses. The predictive value of G×E and parental myopia was estimated using receiver operating characteristic curves. Myopia prevalence was 12%. Both GRS (*P *< 0.01) and ERS (*P *< 0.01) were significantly associated with myopia and AL/CR, as was G×E interaction (*P *< 0.01 for myopia; *P *= 0.07 for AL/CR). The predictive value of parental myopia was 0.67 (95% CI 0.65–0.70), similar to the values of GRS (0.67; 95% CI 0.64–0.70; *P *= 0.98) and ERS (0.69; 95% CI 0.66–0.72; *P *= 0.98). Adding G×E interaction significantly improved the predictive value to 0.73 (95% CI 0.70–0.75; *P *< 0.01). This study provides evidence that nature and nurture are equally important for myopia and AL/CR; however, the combination has the strongest influence. Since myopia genes are common in the population, adjustment of lifestyle should be a major focus in the prevention of myopia.

**Electronic supplementary material:**

The online version of this article (10.1007/s10654-019-00512-7) contains supplementary material, which is available to authorized users.

## Introduction

Myopia is the most common eye disorder in developed countries. Around 50% of young adults in Europe and up to 83% of the Chinese university students have myopia [1, 2]. The global myopia prevalence is rising and expected to increase from one in three persons in 2000 to half of the worldwide population in 2050 [3]. Myopia is caused by an axial elongation of the eye accompanied by structural changes of the retina and choroid. Although myopia can be optically corrected, it is associated with an increased risk of visual impairment and blindness later in life due to retinal complications such as myopic macular degeneration, cataract and glaucoma [4]. A higher degree of myopia results in an earlier onset of retinal complications [5].

Myopia is caused by a complex interplay between nature and nurture [6]. Recently, large genome-wide association studies have identified 161 independent loci for refractive error, which explain 8% of the variance of spherical equivalent in adults and can discriminate myopia from hyperopia with a 0.77 accuracy [7, 8]. Established environmental risk factors that have been associated with myopia include extended near work and minimal outdoor exposure [9–11], and lifestyle in childhood is most likely the major cause of the rapid rising prevalence. Whether lifestyle can alter the outcome of a genetic susceptibility for myopia is currently unsettled. Several studies in adults have demonstrated gene-environment interactions for refractive error, in particular with education [12–15]. However, whether this reflects a certain lifestyle in childhood is unclear and G×E interaction studies in children have been limited [13, 14, 16, 17].

Children with an early onset of myopia are most likely to develop high myopia [18, 19]. Postponing myopia onset or, even better, preventing the onset can be achieved by lifestyle factors, such as spending many hours outdoors [20, 21]. As changing habits is extremely difficult [22], knowledge on susceptibility may help children at risk to adhere lifestyle advice. This knowledge may be acquired by assessing parental myopia or calculating a genetic risk score when DNA analysis is feasible [7, 8, 17, 23]. Whether the latter has additional value is currently unknown.

In the Generation R birth cohort, we previously created a prediction model for myopia based on time spent outdoors, sports participation, number of books read per week, time spent reading, parental myopia, and ethnicity [24]. In the current analysis, we implemented known genetic factors to study gene-environment interactions using genetic and environmental risk scores. We also investigated the relationship between parental myopia and genetic and environmental factors and assessed the predictive value of these variables to identify children at risk for early onset myopia.

## Methods

### Study population: generation R

Generation R is a population-based prospective cohort of 9778 pregnant women and their children who were born between April 2002 and January 2006 in Rotterdam, The Netherlands. The exact methodology of the Generation R study has been described elsewhere [25, 26]. Children were invited to the research center at the age 9 years. Of the initial cohort, 5862 (60%) children participated at the age of 9 years. Genetic data was available for 5731 children, and 3422 of them received eye measurements (58%). The study protocol was approved by the Medical Ethical Committee of the Erasmus Medical Centre, Rotterdam (MEC 217.595/2002/20), and conducted according to the Declaration of Helsinki. Written informed consent was obtained from all participants.

### Eye measurements

Automated cycloplegic refraction was performed in a random sample of children (42%). Two drops (three in case of dark irises) of cyclopentolate (1%) with 5 min interval were dispensed at least 30 min before refractive error measurement. Pupil diameter was ≥ 6 mm at the time of measurement. Children with a visual acuity of more than 0.1 logarithm of the minimum angle of resolution at a 3-m distance by means of the Early Treatment Diabetic Retinopathy Study method in at least 1 eye or children with an ophthalmologic history were referred to an ophthalmologist or orthoptist to identify myopia [27]. Children with visual acuity of 0.1 logarithm of the minimym angle of resolution or less or no glasses or ophthalmic history were classified as nonmyopic [28, 29]. Spherical equivalent (SER) was calculated as the sum of the full spherical value and half of the cylindrical value. Myopia was defined as SER ≤ − 0.50 diopter in at least one eye. Since SER was not available for the whole sample, the axial length/corneal radius (AL/CR) ratio was used as a proxy for refractive error. Ocular biometry was measured by Zeiss IOL-master 500 (Carl Zeiss MEDITEC IOL-master, Jena, Germany). For axial length (AL) five measurements per eye were averaged to mean AL. Three measurements of corneal radius (K1 and K2) were taken of both eyes, and mean corneal radius was calculated (CR). Mean AL/CR ratio was calculated by dividing AL (mm) by CR (mm) for both eyes, divided by two.

### Environmental variables

Environmental variables were measured using a questionnaire filled in by the parents when the child was 9 years old. For outdoor exposure, the questions “how many days per week does your child play outside” and “how long does your child approximately play outside per day” were asked. Mean daily outdoor exposure was calculated by multiplying the number of days by time in minutes divided by seven. Walking or cycling to and from school was processed similarly. Outdoor exposure was calculated as the sum of playing outside and walking or cycling to and from school. For computer use and watching television, the question “how much time does your child use the computer/watch television in the morning/afternoon/evening” was asked for weekdays and weekend days separately. Mean daily computer use and watching television was calculated by dividing the total time per week by seven. Time spent reading was asked per week (never, < 5 h/week, 5–10 h/week, 11–15 h/week or > 15 h/week), number of books read per week (< 1 or 1 ≥ per week) and reading distance was asked and categorised in < 30 cm or ≥ 30 cm. For parental myopia, 0, 1 or 2 myopic parents was registered by questionnaire.

### Environmental risk score

Outdoor exposure, books per week, computer use, reading time and watching television were standardized into a mean of 0 en standard deviation of 1. A multivariate regression model including reading distance, outdoor exposure, number of books per week, computer use, time reading and watching television and interaction effects between them were tested. Backward linear regression analyses were performed until the final model only included significant environmental risk factors (*P *< 0.05). Environmental risk scores (ERS) were computed for each individual using the beta-coefficients of the final multivariate regression model multiplied by the standardized values of the risk factors.

### Genotyping and quality control

Genotyping and quality control were performed as described in Medina-Gomez C et al. [30]. In summary, blood samples were taken from cord blood at birth or venepuncture at the age of 6 years during their visit at the research center. Genotyping was performed with the Illumina HumanHap 610 (at birth) or 660 (at age 6 years) Quad Chips. Quality control procedures were performed using PLINK [31]. Filters were used for marker call rate (calling < 0.2 – < 0.05), minor allele frequency (MAF) ≥ 1%, and deviation from Hardy–Weinberg equilibrium (*P *< 10^−6^). Additional quality control steps included checks for excess heterozygosity, sex mismatch, relatedness and missing data.

### Genetic risk scores

GRS were computed using the summary statistics from a large meta-analysis [7]. We incorporated genetic variants with minor allele frequency greater than 1% and an imputation information score greater than 0.5 or minimac R^2^ greater than 0.8. *P* value based clumping was performed in PLINK using one genetic variant per linkage disequilibrium region [32]. Genetic variants with an r-squared smaller than 0.2 and a physical-distance over 500 kb, excluding the major histocompatibility complex region, were selected. For each individual, GRS values were calculated in PLINK across the following strata of *P*-value thresholds: 5.0 × 10^−8^, 5.0 × 10^−7^, 5.0 × 10^−6^, 5.0 × 10^−5^, 5.0 × 10^−4^, 0.005, 0.01, 0.05, 0.1, 0.5, 0.8 and 1.0. The proportion of variance of AL/CR explained by each GRS model was calculated as the difference in the r-squared between two linear regression models: one in which AL/CR was regressed on age, sex and the first ten principal components, and the other also including the GRS as an additional covariate.

### Gene-environment interaction and correlation

Gene-environment interaction (G×E) is defined as a different effect of a genotype on disease risk in persons with different environmental exposures. In contrast, gene-environment correlation (rGE) refers to the association of different genotypes on environments, in other words individuals are selectively exposed to different environments based on their genetics. Presence of rGE could confound G×E interaction analyses and was therefore assessed [33, 34].

### Statistical analyses

Myopia yes/no was considered the dichotomous outcome variable; AL/CR was used as the continuous outcome. Association analyses were based on cross-sectional data, and prevalence odds ratios were used to represent risk of myopia. Participants with myopia were compared to controls with respect to age, sex, ethnicity, AL/CR and environmental factors using t-tests for continuous variables and Chi square tests for binary variables. Missing information on the covariates varied between 0 and 36% (Table 1). Multiple imputation procedures were performed to replace missing covariates for the most likely values to avoid bias in the analyses using multivariate imputations by chained equations (MICE) [35]. GRS and ERS were computed using linear regression analyses and the proportion of phenotypic variance of AL/CR explained was computed using the R^2^ minus the reference model including age, sex and ethnicity and first ten principal components. Linear regression (for AL/CR) and logistic (for myopia) analyses were performed to test for G×E interactions and rGE correlations, adjusted for age, sex and first ten principal components. Sensitivity analyses were performed for G×E interaction restricted to European children to capture ethnicity-related differences in lifestyle risk factors. The predictive value (area under the receiver operating characteristic curve, AUC) of myopia versus no myopia was calculated for parental myopia, ERS, GRS and combinations of them using pROC package in R [36]. All analyses were performed in SPSS software version 24.0 and R statistical software version 1.1.456 [37, 38].

**Table 1:** General characteristics

Generation R cohort (N = 3422)^a^		Missing (%)	Myopia (N = 391)	No myopia (N = 2900)	*P*-value^b^
Myopia (%)	11.9	4	–	–	–
Age (± SD; years)	9.79 (0.34)	0	9.82 (0.36)	9.79 (0.33)	0.05
Sex (% ♀)	50.8	0	51	51	0.41
Ethnicity (% EUR)	87.8	0	78.0	89.3	<0.01
AL/CR (± SD)	2.97 (0.10)	1	3.11 (0.10)	2.95 (0.08)	<0.01
Reading distance (% < 30 cm)	49.1	36	66.9	46.6	<0.01
Outdoor exposure (hr/day)	1.09 (0.75)	17	1.02 (0.73)	1.09 (0.75)	0.10
Books per week (% > 1)	44.6	32	57.3	43.0	<0.01
Computer use (hr/day)	0.72 (0.78)	20	0.79 (0.90)	0.72 (0.77)	0.16
Time reading (% > 5 h/wk)	38.3	32	46.3	37.1	<0.01
Watching television (hr/day)	1.70 (1.19)	20	1.78 (1.38)	1.69 (1.15)	0.25
Parental myopia (% 1 and % 2)	40.2 15.4	33	44.2 26.5	39.6 14.1	<0.01 <0.01

^a^3406 participants with complete data on AL/CR and 3291 participants with complete data on myopia. Total amount of participants is 3422

^b^P-values are corrected for age and sex

*SD* standard deviation, *EUR* European; *AL*/*CR* axial length corneal curvature ratio; *hr*/*day* average hours per day. Missing information on the variables were imputed using multiple imputations, with the exemption of myopia and AL/CR

## Results

Data from 3422 children entered the analyses and a myopia prevalence of 12% was calculated (Table 1). Children with myopia were more often non-European, had more often a short reading distance, spent more time on reading, had a tendency towards spending less time outdoors and had more often 1 of 2 myopic parents than their peers (Table 1). A backward regression model showed that outdoor exposure (*P *= 0.03), reading distance (*P *< 0.01) and number of books read per week (*P *< 0.01) were significantly associated with AL/CR (Table 2). No significant interactions were found between the environmental variables. ERS explained 1.1% of the variance of AL/CR and 2.1% of myopia (Table 3).

**Table 2:** Full and backward regression model with environmental predictors for AL/CR

N = 3406	Full regression model			Backward regression model		
	Estimate	SE	*P*-value	Estimate	SE	*P*-value
Outdoor exposure^a^	− 0.004	0.002	0.03	− 0.004	0.002	0.03
Reading time^a^	0.003	0.002	0.16	–	–	–
Reading distance^a^	− 0.007	0.002	<0.01	− 0.007	0.002	<0.01
Watching television^a^	0.000	0.002	0.86	–	–	–
Computer use^a^	0.002	0.002	0.30	–	–	–
Books per week^a^	0.005	0.002	0.05	0.006	0.002	<0.01

^a^Environmental variables are standardized and adjusted for age, sex and ethnicity

*Estimate* Beta-coefficient; *SE* standard error

**Table 3:** Variance explained by environmental risk score (ERS), genetic risk score (GRS) and the interaction term (ERS x GRS) for AL/CR and myopia

**AL/CR (N = 3406)**	**Variable**	**Estimate**	**SE**	***P*** **-value**	**Variance explained (%)** ^**f**^
Reference model^a^	NA	NA	NA	NA	1.9
ERS model^b^	ERS	0.010	0.002	<0.01	3.0
GRS model^c^	GRS	0.017	0.002	<0.01	5.0
ERS + GRS model	ERS GRS	0.010 0.017	0.002 0.002	<0.01 <0.01	6.0
ERS x GRS model^d^	ERS GRS ERS x GRS	0.010 0.017 0.004	0.002 0.002 0.002	<0.01 <0.01 0.07	6.1

**Myopia (N = 3291)**	**Variable**	**Odds ratio**	**95% CI**	***P*** **-value**	**Variance explained (%)** ^**f**^
Reference model^a^	NA	NA	NA	NA	1.7
ERS model^b^	ERS	1.048	1.035–1.061	<0.01	3.8
GRS model^c^	GRS	1.045	1.033–1.056	<0.01	4.3
ERS + GRS model^d^	ERS GRS	1.046 1.043	1.033–1.058 1.032–1.055	<0.01 <0.01	6.2
ERS x GRS model^e^	ERS GRS ERS x GRS	1.046 1.043 1.024	1.033–1.058 1.032–1.055 1.008–1.039	<0.01 <0.01 <0.01	6.7

^a^Reference model includes age, sex and ethnicity

^b^ERS adjusted for age, sex and ethnicity

^c^GRS adjusted for age, sex and first 10 principal components

^d^ERS and GRS adjusted for age, sex and first 10 principal components

^e^ERS, GRS and the interaction term ERS x GRS adjusted for age, sex and first 10 principal components

^f^Explained variance is computed as: NagelkerkeR^2^ * 100%

*AL*/*CR* axial length corneal curvature ratio, *ERS* environmental risk score, *GRS* genetic risk score, *Estimate* Beta-coefficient, *SE* standard error, *NA* not applicable, 95% CI = 95% confidence interval

**Fig. 1:**
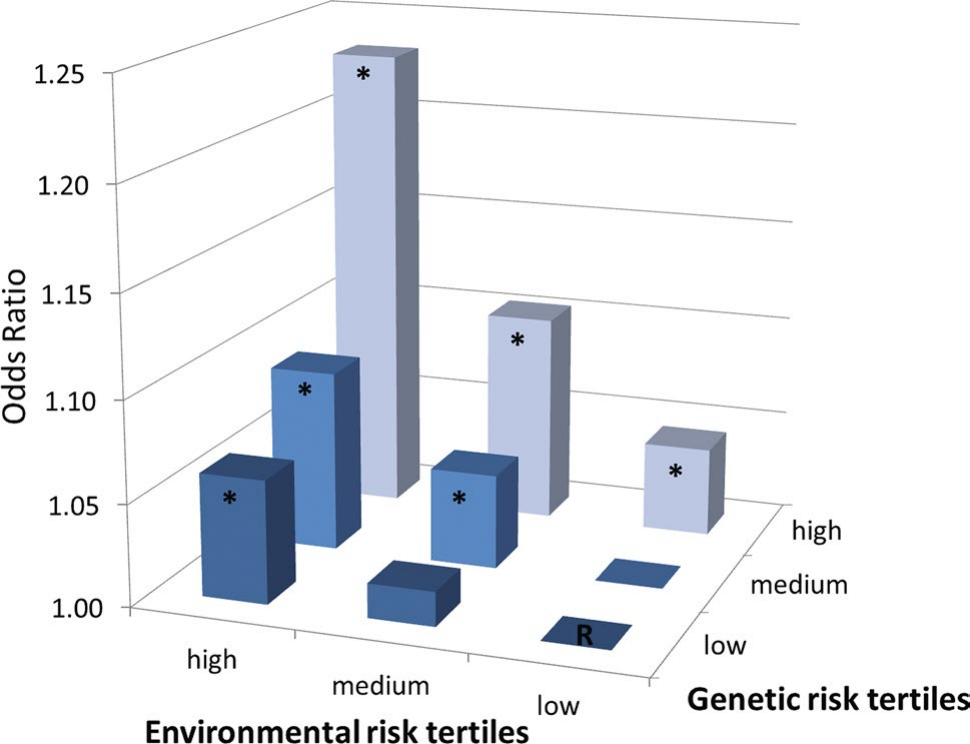
Odds Ratio for myopia per GRS and ERS tertiles

G×E interaction was borderline significant for AL/CR (*P *= 0.07) and significant for myopia (*P *< 0.01), indicating that the effect of GRS on AL/CR and myopia increased within higher levels of ERS (Table 3). Analyses restricted to children with European ancestry showed similar results (*P *= 0.09 for AL/CR and *P *< 0.01 for myopia), indicating that ethnicity-related differences in lifestyle did not bias the G×E results. Figure 1 shows that the risk of myopia among subjects who were in the highest tertiles for GRS and ERS was increased (OR_combined_ = 1.23; 95% CI 1.18–1.29), and was higher than multiplication of the risks among individuals with only one of these factors (OR_combined_ for high GRS = 1.04; 95% CI 1.00–1.09; OR_combined_ for high ERS = 1.06; 95% CI 1.01–1.11) (Table S3, Fig. 1).

A total of 243,261 genetic variants were available for the GRS, which ranged from *P*-value threshold 5.00E − 08 (including 175 variants) to *P*-value threshold 1 (including 243,261 variants). The highest proportion of the variance explained by genetic variants was the stratum of GRS with *P*-value threshold 0.1 (Table S1), which included 65,426 variants for AL/CR (4.4%) and myopia (2.3%). Significant rGE was found from *P*-value threshold 5.00E − 05 onwards (≥ 784 variants) (β = 0.047 to β = 0.062, *P *< 0.01 to *P *= 0.03), meaning this could bias the results of G×E analyses (Table S2). Therefore, GRS including only 175 genome-wide significant variants were used for G×E analyses.

Parental myopia was associated with both ERS (1 myopic parent: β = 0.083, *P *= 0.06; 2 myopic parents: β = 0.160, *P *< 0.01) and GRS (1 myopic parent: β = 0.225, *P *= < 0.01; 2 myopic parents: β = 0.226, *P *< 0.01), indicating that parental myopia comprises shared genetic and environmental factors (Table 4). The prevalence of myopia was 8.3% among children without myopic parents, 13.7% among children with 1 myopic parent, and 18.4% among children with 2 myopic parents (P trend < 0.01). The predictive value (calculated as area under the receiver operating characteristic curve, AUC) for parental myopia was 0.67 (95% CI 0.65–0.70), which was not statistically different from the AUC for GRS (0.67; 95% CI 0.64–0.70; *P *= 0.98) or for ERS (0.69; 95% CI 0.66–0.72; *P *= 0.98). Combining parental myopia with GRS, ERS or G×E, improved the AUC to 0.70, 0.71, and 0.73 respectively (95% CI 0.67–0.73; *P *< 0.01; 95% CI 0.68–0.73; *P *< 0.01; 95% CI 0.70–0.75; *P *< 0.01; Table 5).

**Table 4:** Association between parental myopia and environmental risk score and genetic risk score

N = 3422	Estimate	SE	*P*-value
**ERS model** ^a^			
1 myopic parent	0.083	0.043	0.06
2 myopic parents	0.160	0.067	0.02
**GRS model** ^b^			
1 myopic parent	0.225	0.044	<0.01
2 myopic parents	0.226	0.059	<0.01

^a^ERS adjusted for age, sex and ethnicity

^b^GRS adjusted for age, sex and first 10 principal components

*ERS* environmental risk score, *GRS* genetic risk score, *Estimate* beta-coefficient, *SE* standard error

**Table 5:** The predictive value (area under the receiver operating characteristic curve, AUC) of myopia versus no myopia

N = 3291	AUC	95% CI	*P*-value^c^
Reference model^a^	0.63	0.60–0.66	<0.01
Parental myopia model^b^	0.67	0.65–0.70	–
GRS model^b^	0.67	0.64–0.70	0.78
ERS model^b^	0.69	0.66–0.72	0.98
GRS + parental myopia^b^	0.70	0.67–0.73	<0.01
ERS + parental myopia^b^	0.71	0.68–0.73	<0.01
ERS*GRS + parental myopia^b^	0.73	0.70–0.75	<0.01

^a^Reference model includes age, sex and first ten principal components

^b^Adjusted for age, sex and first ten principal components

^c^In comparison with the parental myopia model

*ERS* Environmental risk score, *GRS* genetic risk score, *AUC* area under de curve; 95% CI = 95% confidence interval

## Discussion

Within our sample of Dutch children aged 9 years old, we found a myopia prevalence of 12%. The risk profile of children who were myopic included high genetic load (high GRS) for myopia and AL/CR, and environmental risk factors such as short reading distance, reading > 1 book per week and < 7 h outdoor exposure per week. Children with a high GRS in combination with high ERS had a greater risk of myopia compared to children with only one of these factors, and this gene-environment interaction was statistically significant. Parental myopia was associated with ERS as well as PRS, indicating shared genetic and shared environmental factors. The predictive value of parental myopia, ERS, and GRS, and G×E combined was 0.73, significantly higher than models with only one of these variables.

Our study had strengths and limitations. Strengths included the large dataset, the extensive evaluation of lifestyle, the thorough genetic screen, and the young age of participants which enabled identification of determinants close to the onset of the trait. Our analyses were performed using continuous variables, which benefitted statistical power for the G×E investigation. Among limitations are the cross-sectional design of our study and the self-report of the environmental risk factors. Future studies incorporating real-time measurements of near work and outdoor exposure will facilitate more accurate evaluation.

Our study investigated the effect of GRS and ERS on myopia outcomes as single exposures as well as the combination. The GRS in this study was based on the stratum of genetic variants which best explained AL/CR and myopia (4.4% and 2.3%, respectively). Our former calculation was based on only 39 SNPs, and explained a much lower variance for AL/CR (0.7% at age 6 years and 3.7% in adults) [39]. Other studies found 0.6% to 1.1% and 2.3% to 2.6% of the variance explained for spherical equivalent at age 7 and 15 years, respectively [17, 40].

With respect to ERS, we found significant associations for outdoor exposure, books per week, and reading distance with AL/CR. Number of books per week was highly correlated with reading time, and the association with the latter disappeared when both variables were included in the model. Watching television was not associated, and computer use appeared weakly associated but failed to reach statistical significance. This is in line with previous findings [9, 11, 24, 41]. Despite the low proportion of variance explained by ERS (2.1% of myopia and 1.1% AL/CR), its predictive value was 0.69, comparable to earlier lifestyle studies in children [23, 24].

Lifestyle can be genetically determined, and vice versa, familial risk can be driven by environmental factors. We tested the association between GRS and ERS, and found a significant correlation when 784 or more genetic variants (P-value threshold 5.00E − 05, Table S2) were included in the GRS. This would imply that lifestyle may be partly genetically determined by variants involved in myopia, i.e. ERS may be a mediator in the association between GRS and myopia outcomes [34]. A recently published paper provided evidence for a genetic correlation between myopia and IQ, and IQ may influence behaviour leading to more near work and less outdoor exposure [42]. This correlation could confound a true G×E association, therefore, we studied the GRS stratum that did not associate with ERS. The results of the G×E analyses show that the effect of GRS on myopia outcomes is influenced by environmental exposure.

Few G×E interactions for myopia were discovered in previous studies. Verhoeven et al. revealed a biological interaction between education and a GRS including 26 genetic variants for myopia [12]. A genome-environment wide interaction study (GEWIS) found interaction between three genetic markers and education in adult Asian populations [15]. A GEWIS for interaction with near work hinted towards an interaction with lifestyle, but the GRS failed to find evidence for interaction with near work or outdoor exposure [15, 17]. Different from our study is that we created a continuous environmental risk score and genetic risk score including 175 variants, while near work and outdoor exposure in previous studies were used as dichotomous variables and individual SNPs or a GRS including 39 variants was used [15, 17].

Parental myopia has been an established risk factor for years. Our study underscores the statistical evidence that parental myopia represents genetic as well environmental risk factors. According to the results of this and other studies, the predictive value for parental myopia (0.67) is as good as GRS (0.67) or ERS (0.69) [23, 43]. To date, genetic testing for young children is not feasible in a clinical setting nor at population level, hence, determination of GRS is unlikely to become a routine procedure. Ascertainment of parental myopia and ERS is much easier to detect children at risk of myopia before the onset. Clinicians encountering myopic parents with young children should raise awareness about prevention of myopia by lifestyle.

In conclusion, our findings add to the evidence that increased near work and lack of outdoor exposure in childhood significantly enhance the effect of myopia genes. Changing children’s lifestyle in this digital era requires action from all of those involved in child raising, starting with increasing awareness by knowledge dissemination.

## Electronic supplementary material

Below is the link to the electronic supplementary material.

Receiver operating characteristic curve (ROC) of myopia versus no myopia (TIFF 25 kb)

Supplementary material 2 (DOCX 17 kb)

Supplementary material 3 (DOCX 16 kb)

Supplementary material 4 (DOCX 17 kb)

## References

[1] Williams KM, Verhoeven VJM, Cumberland P, Bertelsen G, Wolfram C, Buitendijk GHS (2015). Prevalence of refractive error in Europe: the european eye epidemiology (E3) consortium. Eur J Epidemiol.

[2] Wei S, Sun Y, Li S, Hu J, Yang X, Lin C (2018). Refractive errors in University students in Central China: the Anyang University Students Eye Study. Investig Ophthalmol Vis Sci.

[3] Holden BA, Fricke TR, Wilson DA, Jong M, Naidoo KS, Sankaridurg P (2016). Global prevalence of myopia and high myopia and temporal trends from 2000 through 2050. Ophthalmology.

[4] Verhoeven VJM, Wong KT, Buitendijk GHS, Hofman A, Vingerling JR, Klaver CCW (2015). Visual consequences of refractive errors in the general population. Ophthalmology.

[5] Tideman JL, Snabel MC, Tedja MS (2016). Association of axial length with risk of uncorrectable visual impairment for europeans with myopia. JAMA Ophthalmology..

[6] Miraldi Utz V (2017). Nature versus nurture: a systematic approach to elucidate gene–environment interactions in the development of myopic refractive errors. Ophthalmic Genet.

[7] Tedja MS, Wojciechowski R, Hysi PG, Eriksson N, Furlotte NA, Verhoeven VJM (2018). Genome-wide association meta-analysis highlights light-induced signaling as a driver for refractive error. Nat Genet.

[8] Verhoeven VJM, Iglesias AI, Tedja MS, Leeuwen EM, Hysi PG, Wojciechowski R et al. Newly identified genes for refractive error and risk of myopia. Poster session presented at the Association for Research in Vision and Ophthalmology (ARVO) conference; Baltimore, United States2017.

[9] Tideman JWL, Polling JR, Hofman A, Jaddoe VWV, Mackenbach JP, Klaver CCW (2017). Environmental factors explain socioeconomic prevalence differences in myopia in 6-year-old children. Br J Ophthalmol.

[10] Tideman JWL, Polling JR, Voortman T, Jaddoe VWV, Uitterlinden AG, Hofman A (2016). Low serum vitamin D is associated with axial length and risk of myopia in young children. Eur J Epidemiol.

[11] Huang H-M, Chang DS-T, Wu P-C (2015). The association between near work activities and myopia in children—a systematic review and meta-analysis. PLoS ONE.

[12] Verhoeven VJM, Buitendijk GHS, Rivadeneira F, Uitterlinden AG, Vingerling JR, Hofman A (2013). Education influences the role of genetics in myopia. Eur J Epidemiol.

[13] Wojciechowski R, Yee SS, Simpson CL, Bailey-Wilson JE, Stambolian D (2013). Matrix metalloproteinases and educational attainment in refractive error: evidence of gene-environment interactions in the Age-Related Eye Disease Study. Ophthalmology.

[14] Fan Q, Wojciechowski R, Kamran Ikram M, Cheng CY, Chen P, Zhou X (2014). Education influences the association between genetic variants and refractive error: a meta-analysis of five Singapore studies. Hum Mol Genet.

[15] Fan Q, Verhoeven VJ, Wojciechowski R, Barathi VA, Hysi PG, Guggenheim JA (2016). Meta-analysis of gene-environment-wide association scans accounting for education level identifies additional loci for refractive error. Nat Commun..

[16] Tkatchenko AV, Tkatchenko TV, Guggenheim JA, Verhoeven VJM, Hysi PG, Wojciechowski R (2015). APLP2 regulates refractive error and myopia development in mice and humans. PLoS Genet.

[17] Fan Q, Guo X, Tideman JW, Williams KM, Yazar S, Hosseini SM (2016). Childhood gene-environment interactions and age-dependent effects of genetic variants associated with refractive error and myopia: the CREAM Consortium. Sci Rep..

[18] Tideman JWL, Polling JR, Vingerling JR, Jaddoe VWV, Williams C, Guggenheim JA (2018). Axial length growth and the risk of developing myopia in European children. Acta Ophthalmol.

[19] Fledelius HC (2000). Myopia profile in Copenhagen medical students 1996–98. Refractive stability over a century is suggested. Acta Ophthalmol Scand.

[20] Wu P-C, Chen C-T, Lin K-K, Sun C-C, Kuo C-N, Huang H-M (2018). Myopia prevention and outdoor light intensity in a school-based cluster randomized trial. Ophthalmology.

[21] Wu P-C, Tsai C-L, Wu H-L, Yang Y-H, Kuo H-K (2013). Outdoor activity during class recess reduces myopia onset and progression in school children. Ophthalmology.

[22] Ngo CS, Pan C-W, Finkelstein EA, Lee C-F, Wong IB, Ong J (2014). A cluster randomised controlled trial evaluating an incentive-based outdoor physical activity programme to increase outdoor time and prevent myopia in children. Ophthalmic Physiol Opt.

[23] Jones LA, Sinnott LT, Mutti DO, Mitchell GL, Moeschberger ML, Zadnik K (2007). Parental history of myopia, sports and outdoor activities, and future myopia. Investig Ophthalmol Vis Sci.

[24] Tideman JWL, Polling JR, Jaddoe VWV, Vingerling JR, Klaver CCW (2019). Environmental risk factors can reduce axial length elongation and myopia incidence in 6- to 9-year-old children. Ophthalmology.

[25] Kooijman MN, Kruithof CJ, van Duijn CM, Duijts L, Franco OH, van Ijzendoorn MH (2016). The Generation R Study: design and cohort update 2017. Eur J Epidemiol.

[26] Kruithof CJ, Kooijman MN, van Duijn CM, Franco OH, de Jongste JC, Klaver CC (2014). The generation R study: Biobank update 2015. Eur J Epidemiol.

[27] Camparini M, Cassinari P, Ferrigno L, Macaluso C (2001). ETDRS-fast: implementing psychophysical adaptive methods to standardized visual acuity measurement with ETDRS charts. Investig Ophthalmol Vis Sci.

[28] Leone JF, Mitchell P, Morgan IG, Kifley A, Rose KA (2010). Use of visual acuity to screen for significant refractive errors in adolescents: is it reliable?. Arch Ophthalmol.

[29] O’Donoghue L, Rudnicka AR, McClelland JF, Logan NS, Saunders KJ (2012). Visual acuity measures do not reliably detect childhood refractive error—an epidemiological study. PLoS ONE.

[30] Medina-Gomez C, Felix JF, Estrada K, Peters MJ, Herrera L, Kruithof CJ (2015). Challenges in conducting genome-wide association studies in highly admixed multi-ethnic populations: the Generation R Study. Eur J Epidemiol.

[31] Purcell S, Neale B, Todd-Brown K, Thomas L, Ferreira MA, Bender D (2007). PLINK: a tool set for whole-genome association and population-based linkage analyses. Am J Hum Genet.

[32] Chang CC, Chow CC, Tellier LC, Vattikuti S, Purcell SM, Lee JJ (2015). Second-generation PLINK: rising to the challenge of larger and richer datasets. Gigascience..

[33] Jaffee SR, Price TS (2008). Genotype–environment correlations: implications for determining the relationship between environmental exposures and psychiatric illness. Psychiatry..

[34] Ottman R (1996). Gene-environment interaction: definitions and study designs. Prev Med.

[35] van Buuren S, Groothuis-Oudshoorn K (2010). mice: multivariate imputation by chained equations in R. J Stat Softw.

[36] Robin X, Turck N, Hainard A, Tiberti N, Lisacek F, Sanchez JC (2011). pROC: an open-source package for R and S + to analyze and compare ROC curves. BMC Bioinf.

[37] Team RC. R: A language and environment for statistical computing. 3.5.1 ed: R Foundation for Statistical Computing, Vienna, Austria; 2018.

[38] Corp. I. IBM SPSS Statistics for Windows. Version 24.0 ed: Armonk, NY: IBM Corp.; 2016

[39] Tideman JW, Fan Q, Polling JR, Guo X, Yazar S, Khawaja A (2016). When do myopia genes have their effect? Comparison of genetic risks between children and adults. Genet Epidemiol.

[40] Ghorbani Mojarrad N, Williams C, Guggenheim JA (2018). A genetic risk score and number of myopic parents independently predict myopia. Ophthalmic Physiol Opt.

[41] Smaldone G, Campagna O, Pacella F, Pacella E, La Torre G (2015). Computer use and onset of myopia in children: a systematic review. Senses Sci.

[42] Williams KM, Hysi PG, Yonova-Doing E, Mahroo OA, Snieder H, Hammond CJ (2017). Phenotypic and genotypic correlation between myopia and intelligence. Sci Reports..

[43] Zadnik K, Sinnott LT, Cotter SA, Jones-Jordan LA, Kleinstein RN, Manny RE (2015). Prediction of Juvenile-Onset Myopia. JAMA Ophthalmol..

