# Supplementary figures and images for: Interaction between lifestyle and genetic susceptibility in myopia: the Generation R study
Source: Eur J Epidemiol. 2019 Apr 3;34(8):777–84. doi: 10.1007/s10654-019-00512-7 (PMC6602996; doi:10.1007/s10654-019-00512-7)

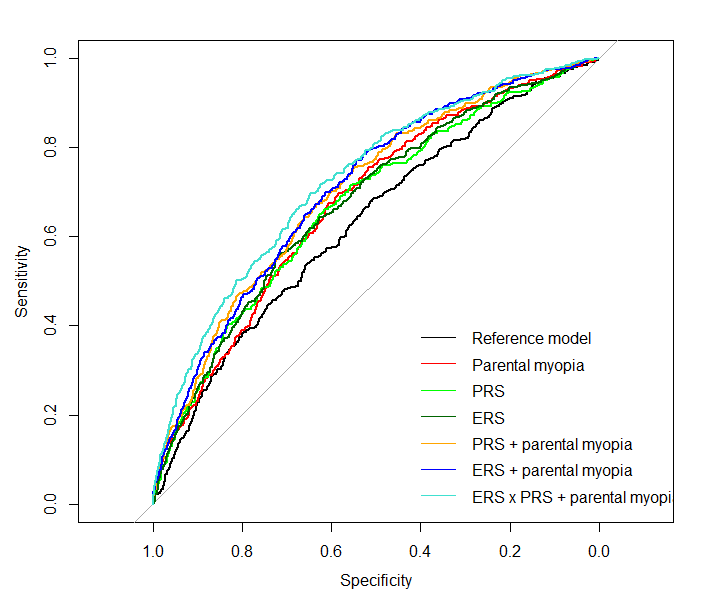

Supplement: Supplementary file 1 — Receiver operating characteristic curve (ROC) of myopia versus no myopia (TIFF 25 kb) [file 10654_2019_512_MOESM1_ESM.tif]
