# Supplementary material for: Interaction between lifestyle and genetic susceptibility in myopia: the Generation R study
Source: Eur J Epidemiol. 2019 Apr 3;34(8):777–84. doi: 10.1007/s10654-019-00512-7 (PMC6602996; doi:10.1007/s10654-019-00512-7)
Supplement: Supplementary file 2 — Supplementary material 2 (DOCX 17 kb) [file 10654_2019_512_MOESM2_ESM.docx]

# Table S1: Variance AL/CR and myopia explained by Genetic risk scores

| Genetic risk score | Meta- GWAS  P-value threshold | N variants in score | Variance explained AL/CR (%)^c^  N=3406 | Variance explained myopia (%)^d^  N=3291 |
| --- | --- | --- | --- | --- |
| Reference model^a^ | NA | NA | 1.9 | 2.4 |
| Score 1^b^ | 5.00E-08 | 175 | 5.4 | 4.3 |
| Score 2 | 5.00E-07 | 248 | 5.3 | 4.3 |
| Score 3 | 5.00E-06 | 404 | 5.6 | 4.4 |
| Score 4 | 5.00E-05 | 784 | 5.4 | 4.4 |
| Score 5 | 5.00E-04 | 2,063 | 5.8 | 4.6 |
| Score 6 | 0.005 | 7,949 | 5.6 | 4.4 |
| Score 7 | 0.01 | 12,657 | 5.6 | 4.4 |
| Score 8 | 0.05 | 39,804 | 6.1 | 4.6 |
| **Score 9** | **0.1** | **65,426** | **6.3** | **4.7** |
| Score 10 | 0.5 | 184,607 | 5.8 | 4.4 |
| Score 11 | 0.8 | 227,764 | 5.8 | 4.5 |
| Score 12 | 1 | 243,261 | 5.7 | 4.5 |

^a^ Reference model includes age, sex and first ten principal components

^b^ Score 1-12: Reference model plus genetic risk scores for different P-value thresholds

^c^ Explained variance is computed as: R² * 100%

^d^ Explained variance is computed as: Nagelkerke R² * 100%

NA = not applicable
