# Supplementary material for: Interaction between lifestyle and genetic susceptibility in myopia: the Generation R study
Source: Eur J Epidemiol. 2019 Apr 3;34(8):777–84. doi: 10.1007/s10654-019-00512-7 (PMC6602996; doi:10.1007/s10654-019-00512-7)
Supplement: Supplementary file 3 — Supplementary material 3 (DOCX 16 kb) [file 10654_2019_512_MOESM3_ESM.docx]

# Table S2: Gene-Environment Correlations

| N=3406 | Effect of GRS on ERS ^a^ | | |
| --- | --- | --- | --- |
| GRS thresholds | **Estimate** | **SE** | **P-value** |
| 5.00E-08 | 0.031 | 0.022 | 0.16 |
| 5.00E-07 | 0.027 | 0.022 | 0.23 |
| 5.00E-06 | 0.035 | 0.020 | 0.09 |
| 5.00E-05 | 0.047 | 0.021 | 0.03 |
| 5.00E-04 | 0.058 | 0.021 | <0.01 |
| 0.005 | 0.062 | 0.023 | <0.01 |
| 0.01 | 0.053 | 0.023 | 0.02 |
| 0.05 | 0.056 | 0.021 | <0.01 |
| 0.1 | 0.059 | 0.021 | <0.01 |
| 0.5 | 0.055 | 0.023 | 0.02 |
| 0.8 | 0.056 | 0.023 | 0.01 |
| 1 | 0.056 | 0.023 | 0.01 |

^a^ Environmental risk scores (ERS) and genetic risk scores (GRS) are standardized and adjusted for age, sex and first ten principal components

ERS = Environmental risk score; GRS = Genetic risk score; Estimate = Beta-coefficient; SE = standard error.
