# Supplementary material for: Interaction between lifestyle and genetic susceptibility in myopia: the Generation R study
Source: Eur J Epidemiol. 2019 Apr 3;34(8):777–84. doi: 10.1007/s10654-019-00512-7 (PMC6602996; doi:10.1007/s10654-019-00512-7)
Supplement: Supplementary file 4 — Supplementary material 4 (DOCX 17 kb) [file 10654_2019_512_MOESM4_ESM.docx]

# Table S3: Odds Ratios per risk score group for myopia

| N=3291 | OR^a^ | 95% CI | P-value | % myopes |
| --- | --- | --- | --- | --- |
| Low GRS – low ERS | 1.000 | - | - | 5.2 |
| Low GRS –medium ERS | 1.017 | 0.973-1.063 | 0.46 | 6.9 |
| Low GRS – high ERS | 1.061 | 1.012-1.111 | 0.01 | 12.2 |
| Medium GRS – low ERS | 0.999 | 0.955-1.045 | 0.95 | 5.7 |
| Medium GRS – medium ERS | 1.047 | 1.000-1.095 | 0.05 | 10.0 |
| Medium GRS – high ERS | 1.089 | 1.041-1.140 | <0.01 | 14.5 |
| High GRS – low ERS | 1.044 | 0.997-1.093 | 0.07 | 9.7 |
| High GRS – medium ERS | 1.103 | 1.054-1.154 | <0.01 | 15.3 |
| High GRS – high ERS | 1.232 | 1.177-1.288 | <0.01 | 27.4 |

^a^ Adjusted for age, sex and first ten principal components

The odds ratio for myopia versus no myopia for environmental risk score tertiles (low, medium or high) and genetic risk score tertiles (low, medium or high). The group with low environmental risk and low genetic risk served as the reference (i.e. OR = 1.0). OR = odds ratio, 95% CI = 95% Confidence Interval
